# Short-term Prophylaxis for Delivery in Pregnant Women with Hereditary Angioedema with Normal C1-Inhibitor

**DOI:** 10.1055/s-0040-1718955

**Published:** 2020-12-21

**Authors:** Caroline Guth de Freitas Batista de Moraes, Liya Regina Mikami, Lilian Pereira Ferrari, João Bosco Pesquero, Herberto José Chong-Neto, Nelson Augusto Rosario Filho

**Affiliations:** 1 Universidade Federal do Paraná, Curitiba, PR, Brazil; 2 Centro Universitário Autônomo do Brasil, Curitiba, PR, Brazil; 3 Universidade Federal de São Paulo, São Paulo, SP, Brazil

**Keywords:** angioedema, estrogens, edema, pregnancy, prophylaxis, angioedema, estrogênios, edema, gravidez, profilaxia

## Abstract

**Objective** To verify the efficacy of short-term prophylaxis for vaginal or cesarean section childbirth with plasma-derived C1-inhibitor concentrate in pregnant women. They should have hereditary angioedema (HAE) and normal plasma C1-inhibitor.

**Methods** Case report of pregnant women diagnosed with HAE with normal C1-inhibitor who had been treated with intravenous C1-inhibitor concentrate for prophylaxis of angioedema attacks when hospitalized for delivery. The exon 9 of the Factor 12 ( *F12* ) genotyping gene was performed by automatic sequencing in all patients.

**Results** Three cases of pregnant women with HAE with normal serum level of C1-inhibitor are reported. The genetic test detected the presence of a pathogenic mutation in the *F12* gene. Deliveries occurred uneventfully and patients had no HAE symptoms in the following 72 hours.

**Conclusion** C1-inhibitor concentrate could be useful to prevent angioedema attacks during and after delivery.

## Resumo

**Objetivo** Verificar a eficácia da profilaxia de curto prazo para o parto vaginal ou cesáreo com inibidor de C1 derivado de plasma concentrado em mulheres grávidas. Eles devem ter angioedema hereditário e inibidor normal de C1 no plasma.

**Métodos** Relato de caso de gestantes diagnosticadas com angioedema hereditário com inibidor de C1 normal que foram tratadas com inibidor intravenoso de concentrado de C1 para profilaxia de ataques de angioedema quando hospitalizadas para o parto. O exon 9 do gene de genotipagem do fator 12 ( *F12* ) foi realizado por sequenciamento automático em todos os pacientes.

**Resultados** Três casos de gestantes com angioedema hereditário com nível sérico normal de inibidor de C1 são relatados. O teste genético detectou a presença de uma mutação patogênica no gene *F12* . Os partos ocorreram sem intercorrências e as pacientes não apresentaram sintomas hereditários de angioedema nas 72 horas seguintes.

**Conclusão** O concentrado de inibidor de C1 pode ser útil para prevenir ataques de angioedema durante e após o parto.

## Introduction

Hereditary angioedema (HAE) is a rare disease, and its prevalence is estimated to be ∼ 1:50,000 inhabitants. It is a genetic disorder of autosomal dominant inheritance. It is defined by the quantitative and/or functional deficiency of C1 esterase inhibitor (C1-INH), or with normal C1-INH and alteration in genes encoding Hageman factor XII ( *FXII* ) of the blood coagulation cascade. 1 2 It leads to edema attacks in the skin and submucosa, in the regions of the face, extremities, genitalia, oropharynx, larynx, tongue, airway and gastrointestinal tract with risk of death from airway obstruction. 1 2

The association between the disease and the estrogen hormone is the main feature of HAE with normal C1-INH. Elevated levels of this hormone in pregnancy, or the use of oral contraceptives, stress and menstrual cycles are triggers of this type of HAE. 1 3 4 Thus, symptoms may become more frequent and severe during pregnancy, delivery, postpartum and lactation in women with HAE. 5 6

Pregnant women with HAE with low level or functional deficiency of C1-INH should be treated with plasma-derived C1 inhibitor concentrate until 6 hours before delivery and could be repeated as needed, as well as 72 hours after the delivery. 1 The hospital where the delivery will take place should have short-term preventive medications such as plasma-derived C1 inhibitor concentrate and trained personnel for the care of patients with HAE. 4 5 6

There is no data regarding plasma-derived C1 inhibitor concentrate short-term prophylaxis for HAE with normal C1-INH. The objective of the present study was to verify the efficacy of short-term prophylaxis of attacks in vaginal or cesarean delivery with plasma-derived C1-inhibitor concentrate in three pregnant women with HAE and normal C1-inhibitor confirmed by molecular mutation analysis of the *F12* gene.

## Description of Cases

We report three pregnant patients diagnosed with HAE and *F12* gene mutation attending the Immunology Division of the Hospital de Clínicas of the Universidade Federal do Paraná in 2018. All three had normal serum levels of C1-INH and C4. Of these, 2 are first cousins (patients 1 and 2) and they all agreed to participate in the present study and signed the informed consent form. Exon 9 genotyping, as well as its flanking regions and splicing sites of the *F12* gene, was performed by automated sequencing on an ABI 3500 Genetic Analyzer sequencer (Applied Biosystems, Foster City, CA, USA).

### Patient 1

Female, 31 years old, primiparous. She reports clinical signs of HAE for 9 years, usually marked lip and facial edema, requiring medical attention and treated with corticosteroids, adrenaline and antihistamines. She did not identify any triggering factors.

Menarche at 15 years old, and she started contraception at 19 years old. At 29 years old, she became pregnant and reported left lower eyelid edema, lasting 5 hours and without treatment, sagging spontaneously.

Mutation search for the factor XII *F12* gene revealed the presence of the c.983C > A mutation in pathogenic heterozygosis (p.Thr328Lys) and confirmed the hypothesis of HAE without C1-INH deficiency.

Plasma-derived C1 esterase inhibitor, 1,000 IU, was administered intravenously 6 hours before cesarean section for short-term prophylaxis at delivery and postpartum. There were no complications during the surgical procedure and within 72 hours, subsequently.

### Patient 2

Female, 44 years old, menarche at 12 years old. She reported lip and facial edema in the first pregnancy, 22 years ago. After this period, she had monthly angioedema attacks in the extremities and abdominal pain. During the attacks, she was treated with adrenaline and corticosteroids, unsuccessfully. The contraceptive was the only medication for continuous use. At 38 years old, she had severe upper airway edema requiring orotracheal intubation and hospitalization for 4 days, and no triggers could be pointed out.

A search for mutation of factor XII *F12* gene confirmed the presence of the mutation c.983C > A in pathogenic heterozygosis (p.Thr328Lys) and confirmed HAE with no C1-INH deficiency.

When she was 43 years old, at the end of the pregnancy, she reported edema in the hands and legs. Fetal echocardiography showed subcutaneous edema and increased echogenicity in intestinal loops with increased peristalsis. The patient was hospitalized for dyspnea and successfully treated with fresh plasma.

**Fig. 1:**
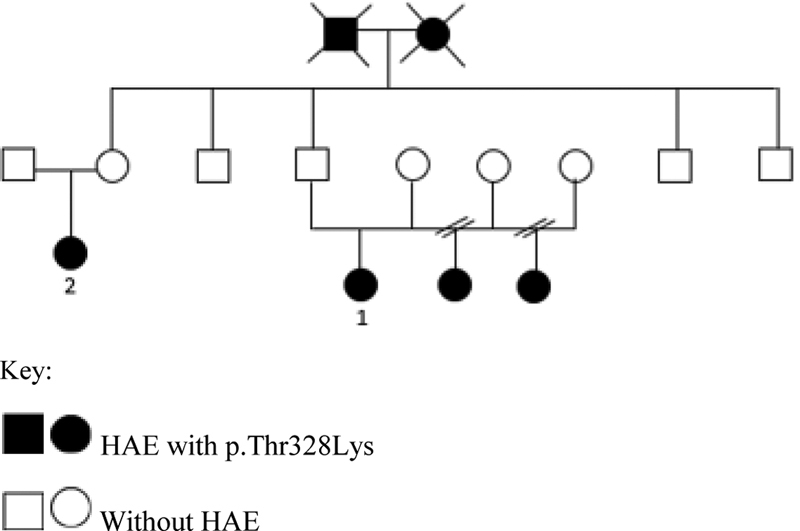
Family Heredogram (Patients 1 and 2).

The cesarean section at the 39 ^th^ gestational week was performed for fetal malformation. The patient received 1,000 IU of intravenous C1-INH inhibitor 1 hour before the cesarean section without complications during the procedure and 72 hours postpartum ( Fig. 1 ).

### Patient 03

Female, 20 years old, primiparous, single HAE case in the family. Menarche at 11 years old. Abdominal pain began in childhood and, from the age of 15 years old, monthly edema attacks of lips, tongue, face, eyelids, hands, and feet lasting from 3 to 4 days without improvement and no response to adrenaline and corticosteroids. She was treated with fresh plasma with improved outcomes.

Mutation search for the factor XII *F12* gene confirmed the presence of the mutation c.983C > A in pathogenic heterozygosis (p.Thr328Lys) and confirmed HAE without C1-INH deficiency.

She got pregnant at 19 years old and reported 2 episodes of foot edema and mild lip edema, without medical complications, improving in 12 hours. The patient received intravenous C1 inhibitor, 1,000 IU, 1 hour before vaginal delivery for short-term prophylaxis. During the labor and postpartum, the patient was in good general condition, and 2 days after the delivery she had edema attacks in her left hand ( Table 1 ).

**Table 1:** Laboratory workout

	Case 1	Case 2	Case 3	Reference values
C4 Complement Fraction (mg/dL)	22	27	19	10–40
Quantitative C1 Inhibitor (mg/dL)	26	20	28.8	21–39
Functional C1 Inhibitor (%)	81	85	71	70–130

## Discussion

Hereditary angioedema with normal C1-INH was first described in the year 2000. 6 As noted, it is rare, mainly affects women, and is characterized by normal C1-INH levels and activities 7 and by mutations in the *F12* gene. 8

A predominance in females is associated with the estrogen hormone, a hallmark of HAE with normal C1-INH. 5 7 Estrogen has a regulatory role in the synthesis of FXII protein, as well as of several genes and proteins of the coagulation cascade and of the kallikrein-kinin system, increasing synthesis of bradykinin, kallikrein, vascular permeability and consequently causing edema. 9 This association is important in worsening the attacks in women, ranging from childhood, puberty, menses, pregnancy and menopause. 1

Short-term prophylaxis with C1-INH concentrate administration up to 6 hours before the procedure or having a dose of C1-INH concentrate available in the delivery room is recommended to prevent a possible bout of edema during childbirth in women with HAE with C1-INH deficiency, but not for HAE with normal C1-INH. 3 7

The present report of three pregnant patients demonstrated the efficacy and safety of plasma-derived C1-INH as short-term prophylaxis for HAE with normal C1-INH. There was no severe edema during vaginal delivery and/or cesarean section after the intravenous use of C1-INH concentrate. Surgical stress and mechanical abdominal trauma for cesarean delivery or genital mechanical trauma for normal delivery may be triggering factors for HAE. 10

In the postpartum period, special attention should be given to edema triggers. 10 Postpartum seizures usually occur within 72 hours of delivery and can have serious consequences. 1 Patients show different symptoms in the attacks (left lower eyelid edema, left hand edema, vaginal bleeding and hypotension) starting 48 hours after delivery, demonstrating the need for protective medication immediately after delivery. 7 C1-INH concentrate shortens the duration of attacks by about one third and also reduces the time for the onset of symptom relief. 11

The reported cases bring to light the discussion of preventive therapy of a complex and serious situation, which is the occurrence of HAE attacks during pregnancy and childbirth. Although used in a minority of cases, these should be adequately selected and their diagnosis confirmed by molecular tools. C1-INH concentrate can produce satisfactory results in symptomatic relief and improvement of the quality of life of the patients, especially postpartum. In Brazil, C1-INH concentrate is approved; however, it is not yet included in the list of high-cost drugs provided by the government and only by demand of judicial request to the public health system. 1

## Conclusion

In conclusion, short-term prophylaxis using C1-INH concentrate in vaginal or cesarean delivery could be useful in pregnant patients with HAE and normal C1-INH. Larger studies may verify how C1-INH prevents attacks of HAE in pregnant women with HAE and normal C1-INH.
